# Timosaponin B-II alleviates osteoarthritis-related inflammation and extracellular matrix degradation through inhibition of mitogen-activated protein kinases and nuclear factor-κB pathways in vitro

**DOI:** 10.1080/21655979.2021.2024685

**Published:** 2022-01-30

**Authors:** Xinwei Liu, Dulei Xiang, Wenming Jin, Gen Zhao, Han Li, Bing Xie, Xiaochuan Gu

**Affiliations:** a Department of Orthopaedics, General Hospital of Northern Theater Command, Shenyang People’s Republic of China; b Graduate School, Jinzhou Medical University, Jinzhou, People’s Republic of China; c Graduate School, China Medical University, Shenyang, People’s Republic of China; d Graduate School, Dalian Medical University, Dalian, People’s Republic of China; e Department of Orthopedics, Changhai Hospital, Navy Medical University, Shanghai, People’s Republic of China

**Keywords:** Osteoarthritis, inflammation, extracellular matrix degradation, Timosaponin B-II

## Abstract

Osteoarthritis (OA), an inflammatory response in chondrocytes, leads to extracellular matrix (ECM) degradation and cartilage destruction. Timosaponin B-II (TB-II) is the main bioactive component of Rhizoma Anemarrhenae with reported antioxidant and anti-inflammatory effects. This study investigated the anti-OA function and mechanism of TB-II on IL-1β-stimulated SW1353 cells and primary rat chondrocytes. We firstly screened the concentration of TB-II in SW1353 cells and primary rat chondrocytes using CCK-8 assay. Thereafter, SW1353 cells and chondrocytes were, respectively, pretreated with TB-II (20 and 40 μg/mL) and TB-II (10 and 30 μg/mL) for 24 h and then stimulated with interleukin 1β (IL-1β, 10 ng/mL) for another 24 hours. Results showed that TB-II suppressed the production of reactive oxygen species, the protein levels of inducible nitric oxide synthase and cyclooxygenase-2 in IL-1β-stimulated SW1353 cells and chondrocytes. IL-1β-induced high secretion levels of nitric oxide and prostaglandin 2, TNF-α, IL-6 and MCP-1 were down-regulated by TB-II treatment, indicating an anti-inflammatory effect of TB-II on OA *in vitro* condition. Moreover, TB-II weakened the mRNA and protein expression of (matrix metalloproteinase) MMPs including MMP-1, MMP-3, and MMP-13, indicating the protection of TB-II against ECM degradation. Mechanically, TB-II suppressed MAPKs and NF-κB pathways under IL-1β stimulation evidenced by the down-regulated protein expression of p-ERK, p-p38, p-JNK, p-p65 and the reduced translocation of p65 subunit to the nucleus. The present study demonstrated that TB-II might become a novel therapeutic agent for OA treatment through repressing IL-1β-stimulated inflammation, oxidative stress and ECM degradation via suppressing the MAPKs and NF-κB pathways.

## Graphical abstract

**Figure:**
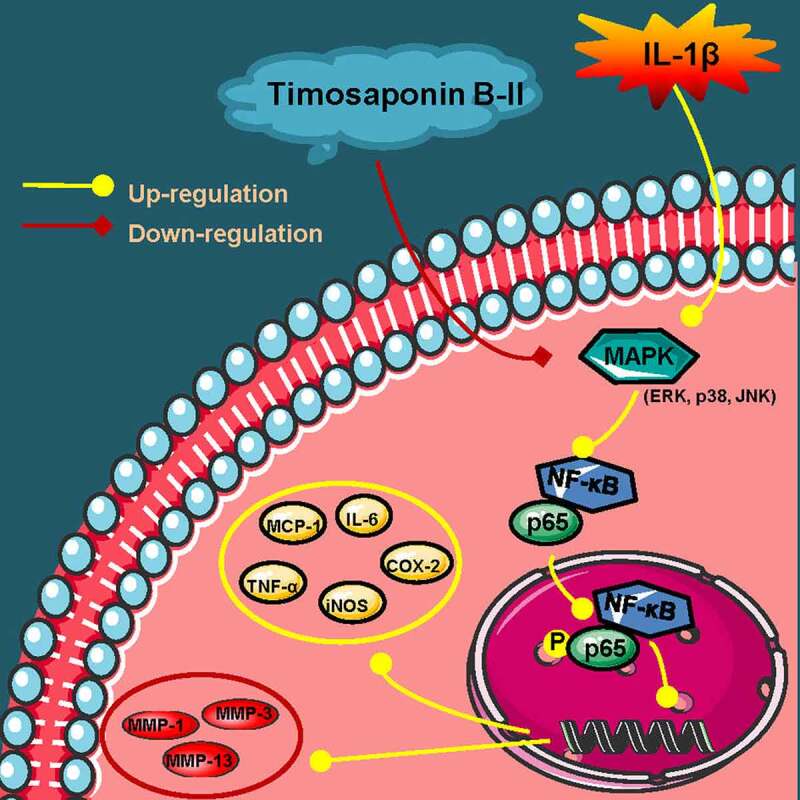

## Introduction

Osteoarthritis (OA) is characterized by extracellular matrix (ECM) degradation and cartilage tissue destructions. As the specific pathogenesis of OA is still a mystery, there is currently no effective clinical intervention. OA has seriously affected the life quality of patients and increased the social burden. Recent studies have reported that the balance of anabolism and catabolism of ECM is closely related to OA [1,2]. ECM, composed of aggrecan and type II collagen (COL-II), is able to maintain the stability of the intracellular environment and the cartilage structure [1,3]. Moreover, ECM can realize the long-term weight-bearing capacity of the joints due to its unique fluid pressurizing function [1]. During the progression of OA, excessive cartilage degrading enzymes such as matrix metalloproteinases (MMPs) were produced by chondrocytes to degrade COL-II and accelerate the decomposition of ECM [4].

Additionally, several studies have shown that inflammatory response leads to OA, affecting the process of cartilage degradation [5]. The tumor necrosis factor α (TNF-α) and pro-inflammatory cytokines interleukin 1β (IL-1β) increase the catabolic activity of chondrocytes, leading to the production of proteolytic enzymes such as MMPs [6]. Recent researches have shown that IL-1β can significantly up-regulate the production of nitric oxide (NO), inducible nitric oxide synthase (iNOS), interleukin 6 (IL-6), prostaglandin 2 (PGE_2_) and cyclooxygenase 2 (COX-2), followed by aggravating inflammatory effects and OA progression [5–7]. Furthermore, the imbalance in reactive oxygen species (ROS) formation and clearance leads to increased expression of inflammatory cytokines and chemokines in OA [8]. Therefore, finding effective anti-inflammatory and anti-oxidative drugs to alleviate the development of OA is of great significance.

As there are short of effective drugs for OA, exploring traditional Chinese medicine with anti-inflammatory and anti-oxidant properties might be novel direction for OA treatment. Timosaponin B-II (TB-II) is the main and effective bioactive component of Rhizoma Anemarrhenae. Rhizoma Anemarrhenae is also known as Zhi-Mu in Chinese, the dried rhizome of Anemarrhena asphodeloides Bge, is a traditional Chinese herbal medicine in China and other East Asian countries [9]. Several types of research have exhibited that TB-II promotes to scavenge oxygen-free radicals and inhibits the secretion of inflammatory cytokines [10–12]. In addition, TB-II can also promote osteoblast proliferation and bone calcification [13]. An appropriate amount of TB-II can prevent bone loss in rats suffered ovariectomy via promoting bone formation [14]. These data suggest the important function of TB-II in bone-related diseases. However, the effects of TB-II in OA has not been studied before.

In this study, we hypothesized that TB-II might exert the anti-inflammatory and anti-oxidant functions on the development of OA. We sought to investigate the effects of TB-II as well as its involving mechanism in chondrosarcoma SW1353 cells and primary rat chondrocytes, mimicking the osteoarthritis condition in vitro. Herein, our findings confirmed that TB-II inhibited IL-1β-induced inflammation and ECM degradation via repressing the MAPKs and NF-κB signaling pathways.

## Materials and methods

### Cell culture

Chondrosarcoma cells (SW1353, Zhong Qiao Xin Zhou, Shanghai, China) were cultured in L-15 Dulbecco’s modified Eagle’s medium (PM151010, Procell, Wuhan, China) containing 10% fetal bovine serum (04-011-1A, BI,Israel). Primary rat chondrocytes (iCell, Shanghai, China, cell identification shown in Figure S1) were cultured in ICell Primary Chondrocyte Culture System (PriMed-iCell-020, iCell). Then, all cells were maintained at 37 °C in an incubator with 5% CO_2_.

### Cell Counting Kit-8 (CCK-8) assay

The effect of TB-II (IT0640, Solarbio, China) on cell inhibition and viability were determined using CCK-8 assay. Cells were cultured in a 96-well plate at a density of 3 × 10^3^ per well. To screen the suitable concentration, SW1353 cells were, respectively, pre-incubated with different concentrations of TB-II (0, 10, 20, 40, 50, 75 and 100 μg/mL), and chondrocytes were, respectively, pre-incubated with different concentrations of TB-II (0, 10, 20, 30, 40, 50 and 75 μg/mL) for 24 h. After that, cells were treated as shown in *Cell culture and treatment* for detecting cell viability. Then the cells were added with 10 μL CCK-8 (96,992, Sigma, USA) and incubated at 37°C for 2 h. The optical density (OD) was read at a wavelength of 450 nm with a microplate reader (Leica Microsystems, Germany). The cell inhibition was obtained by the following formula [15]:

$$cellinhibition=\left[\frac{OD\left(Control\right)-OD\left(Sample\right)}{OD\left(Control\right)}\right]\times 100\%$$

### Cell treatment with IL-1β or TB-II

According to the results of the CCK8 assay, SW1353 cells and chondrocytes were, respectively, pretreated with TB-II (20 and 40 μg/mL) and TB-II (10 and 30 μg/mL) for 24 h and then stimulated with IL-1β (10 ng/mL) for another 24 h. The concentration of IL-1β used was based on a previous report [16]. The cells in the IL-1β group were stimulated with IL-1β (10 ng/mL) for 24 h without TB-II pretreated. The cells in the control group were treated with vehicle containing the solvent of TB-II and the buffer solution of IL-1β.

### Intracellular reactive oxygen species (ROS) detection

An oxidation-sensitive fluorescent probe DCFH-DA (KGT010, KeyGEN, China) was implied to measure the level of intracellular ROS as previously described [17]. Briefly, after treatment, cells were collected and incubated in a serum-free medium containing DCFH-DA at 37 °C for 30 min according to the instructions. ROS level was then detected by NovoCyte flow cytometer (NovoCyte, ACEA, USA).

### Quantitative real-time PCR (qRT-PCR)

qRT-PCR was performed according to a previous study [18]. Briefly, total RNA was isolated from cells using RNA simple Total RNA Kit (DP419, TIANGEN, China). After RNA concentration determination, reverse transcription of RNA into first-strand cDNA was performed using M-MLV Reverse Transcriptase (NG212, TIANGEN). qRT-PCR was performed using SYBR Green Master Mix (SY1020, Solarbio) and 2× Taq PCR Master Mix (KT201, TIANGEN) in the Exicycler^TM^ 96 Real-time Quantitative Thermal Block (BIONEER, Korea). The level of target mRNA was analyzed in a 2^−ΔΔCT^ method and normalized to GAPDH expression. The primer sequences are shown in Table 1.

**Table 1.** Primers for real-time PCR

Gene name	Forward primer (5’‑3’)	Reverse primer (5’‑3’)	Product size (bp)
Human			
COX-2	TTGGGTGTCAAAGGTAA	GATGCGTGAAGTGCTG	136
iNOS	AGCGGTAACAAAGGAGATAG	GGGAACACGGTGATGG	243
MMP-1	TTGGGCTGAAAGTGAC	CAAATCTGGCGTGTAA	164
MMP-3	AGTTTGCTCAGCCTATC	AGAGTGTCGGAGTCCAG	213
MMP-13	TTCCAAAGGCTACAACT	GGTAATGGCATCAAGG	253
Rat			
COX-2	GAACACGGACTTGCTCACTT	ACGATGTGTAAGGTTTCAGG	187
iNOS	TTGGAGCGAGTTGTGGATTG	GTGAGGGCTTGCCTGAGTGA	125
MMP-1	GCAGGTTCTACATTCGTG	ACTTCATAAGCAGCATCA	106
MMP-3	GCTCATCCTACCCATTGCA	GTGTTCGAGTCCAGCTTCC	199
MMP-13	GACCCAGCCCTATCCCT	ACCCTCCATAATGTCATACCC	243

### NO assay

NO levels in the cell supernatant were assessed using NO assay kit (A013, Nanjing Jiancheng Bioengineering Institute, China). The OD value was read at 550 nm on a microplate reader.

### ELISA kit assay

After extracting the cell supernatant, the levels of PGE_2_, TNF-α, IL-6, and MCP-1 were detected using PGE_2_ ELISA kit (EK8103, Liankebio, China and ER1800, Wuhan Fine Biotech Co., Ltd., China), TNF-α ELISA kit (EK182 and EK382, Liankebio, China), IL-6 ELISA kit (EK106 and EK306, Liankebio, China) and MCP-1 ELISA kit (EK187 and EK387, Liankebio, China), respectively, according to the instructions.

### Western blot

Western blot assay was performed as described by Sale et al [19]. Total proteins were extracted from cells using RIPA lysate (R0010, Solarbio) containing phenylmethylsulfonyl fluoride protease inhibitor (PMSF, P0100, Solarbio). Then, protein concentration was detected with a BCA kit (PC0020, Solarbio). After separated by 5-10% SDS-PAGE, transferred to the PVDF membrane, blocked by nonfat milk (A600669, Sangon Biotech, China), proteins on the membrane were incubated overnight at 4°C with the primary antibodies: iNOS (1: 1000, A0312, Abclonal, China), COX-2 (1: 2000, A1253, Abclonal), MMP-1 (1: 2000, A1191, Abclonal and 1: 1000, DF6325, Affinity), MMP-3 (1: 2000, A11418, Abclonal), MMP-13 (1: 2000, A11148, Abclonal and 1: 1000, AF5355, Affinity), p-ERK1/2 (1: 1000, AF1015, Affinity, China), ERK1/2 (1: 1000, AF0155, Affinity), p-p38 (1: 500, AF4001, Affinity), p38 (1: 500, AF6456, Affinity), p-JNK (1: 1000, AF3318, Affinity), JNK (1: 500, AF6318, Affinity), p-p65 (1: 1000, AF2006, Affinity), p65 (1: 1000, AF5006, Affinity), and GAPDH (1: 10,000, 60,004-1-Ig, Proteintech, China). Next, the membrane was incubated with appropriate secondary antibody. Finally, membranes were detected by Western electrogenerated chemiluminescence (ECL) Substrate (D1010, Solarbio) and the images were analyzed by Gel-pro analyzer software (Media Cybernetics, CA, USA).

### Immunofluorescence staining

After fixed with 4% paraformaldehyde and treated with 0.1% Triton X-100 (ST795, Beyotime, China), SW1353 cells were blocked with goat serum (SL038, Solarbio) for 15 min and incubated with the primary antibody p65 (1: 200) overnight. Later, the cells were incubated with Cy3-labeled goat anti-rabbit IgG (1: 200, A0516, Beyotime) for 1 h. The cell nuclei were stained by DAPI.

### Statistical analysis

Data were analyzed by using the Graphpad Prism 8.0 software (GraphPad Software Inc., La Jolla, CA, USA) and expressed as mean ± standard deviation (SD). One-way analysis of variance (ANOVA) followed by Tukey’s test was used for determining statistically significant differences. Differences were considered statistically significant when *p* < 0.05. All experiments were performed at least three times.

## Results

### Effects of TB-II on the cell viability and ROS production of SW1353 cell and chondrocyte

**Figure 1.**
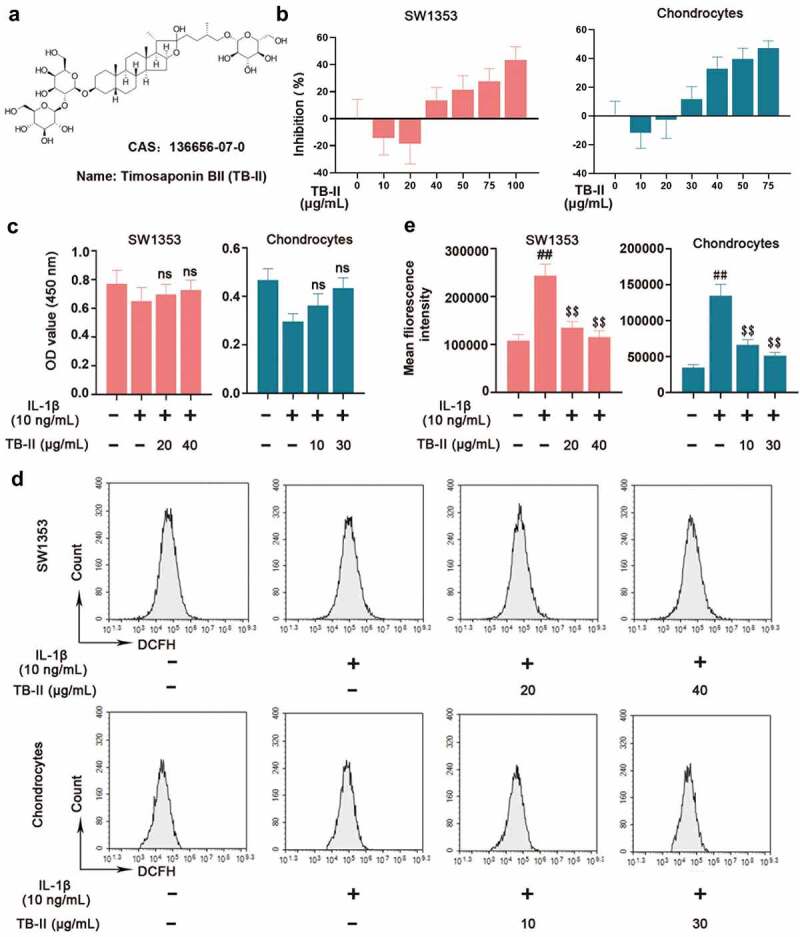
**Effect of Timosaponin BII (TB-II) on IL-1β-induced SW1353 cell and chondrocyte viability and reactive oxygen species (ROS) production**. (a) The chemical structure of TB-II. (b, c) The effect of TB-II on SW1353 cell and chondrocyte viability was determined by the CCK-8 assay. (d, e) ROS levels were detected and quantified. One-way ANOVA, ^##^p < 0.01, compared with control cells; ^$$^p < 0.01, ns: not-significant, compared with IL-1β-treated cells.

To screen out suitable concentration of TB-II, SW1353 cells and chondrocytes were treated with different concentrations of TB-II. The chemical structure of TB-II was shown in Figure 1a. As shown in Figure 1b, TB-II started to promote cell proliferation at 20 μg/mL and inhibit cell proliferation at 40 μg/mL in SW1353 cells, whereas promote cell proliferation at 10 μg/mL and inhibit cell proliferation at 30 μg/mL in chondrocytes. Therefore, 20 μg/mL and 40 μg/mL TB-II were used for subsequent experiments in SW1353 cells. Similarly, 10 μg/mL and 30 μg/mL TB-II were used for subsequent experiments in chondrocytes. Subsequently, SW1353 cells and chondrocytes were pretreated with TB-II (20 and 40 μg/mL) and TB-II (10 and 30 μg/mL) for 24 h, respectively, and then stimulated with IL-1β (10 ng/mL) for another 24 h. The CCK-8 assay showed that TB-II (20 and 40 μg/mL) and TB-II (10 and 30 μg/mL) did not affect SW1353 cell and choncrocyte viability compared with control cells (Figure 1c). Furthermore, flow cytometry was used to detect the ROS level. Our results showed that TB-II treatment reduced ROS production in the IL-1β-induced cells (Figure 1d and e).

### Effects of TB-II on the inflammation in IL-1β-stimulated SW1353 cells and chondrocytes

**Figure 2.**
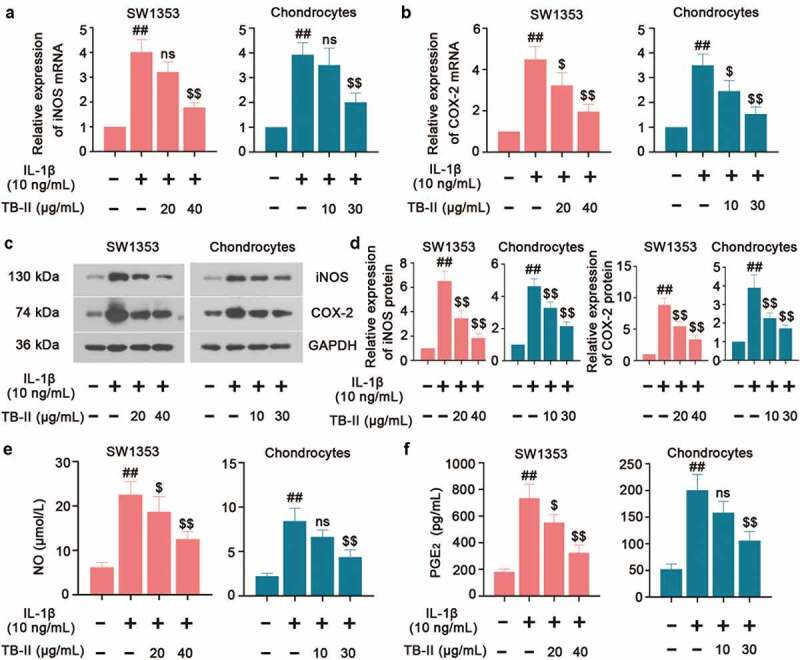
TB-II inhibited the production of oxidant markers in IL-1β-treated SW1353 cells and chondrocytes. The mRNA expression of (a) iNOS and (b) COX-2 were detected by qRT-PCR. (c, d) The protein level of iNOS and COX-2 was detected by Western blot and quantified. GAPDH was conducted as a loading control. The levels of (e) NO and (f) PGE2 were determined using ELISA. One-way ANOVA, ##p < 0.01, compared with control cells; ^$^p < 0.05, ^$$^p < 0.01, ns: not-significant, compared with IL-1β-treated cells.

**Figure 3.**
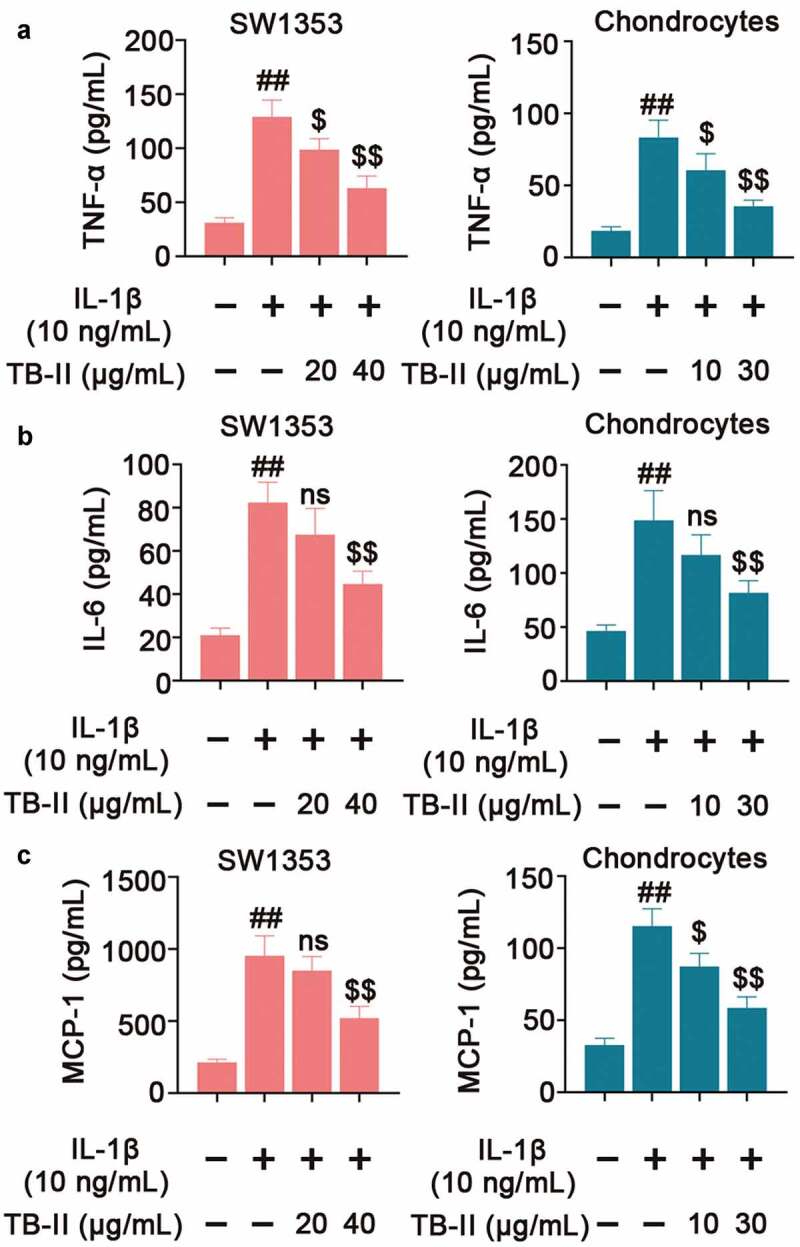
**TB-II inhibited the production of inflammatory cytokines in IL-1β-treated SW1353 cells and chondrocytes**. The levels of (a) TNF-α, (b) IL-6 and (c) MCP-1 were determined using ELISA. One-way ANOVA, ^##^p< 0.01, compared with control cells; ^$^p< 0.05, ^$$^p< 0.01, ns: not-significant, compared with IL-1β-treated cells.

In regard to inflammation, we detected the expression of some pro-inflammatory cytokines. As shown in Figure 2a-D, IL-1β significantly increased the mRNA and protein level of iNOS and COX-2. However, TB-II suppressed IL-1β-induced production of iNOS and COX-2 in both IL-1β-stimulated SW1353 cells and chondrocytes. IL-1β could elevate the production of NO via iNOS activation, and up-regulate the expression of PGE_2_ via stimulating the activity of COX-2. Herein, our results also showed that the expressions of NO and PGE_2_ were obviously increased in IL-1β-induced cells compared with the control cells, whereas TB-II inhibited the increase (Figure 2e and f). Furthermore, IL-1β enhanced the production of TNF-α, IL-6, and MCP-1, but it was reversed by TB-II treatment (Figure 3a-c).

### Effects of TB-II on MMPs production in IL-1β-stimulated SW1353 cells and chondrocytes

**Figure 4.**
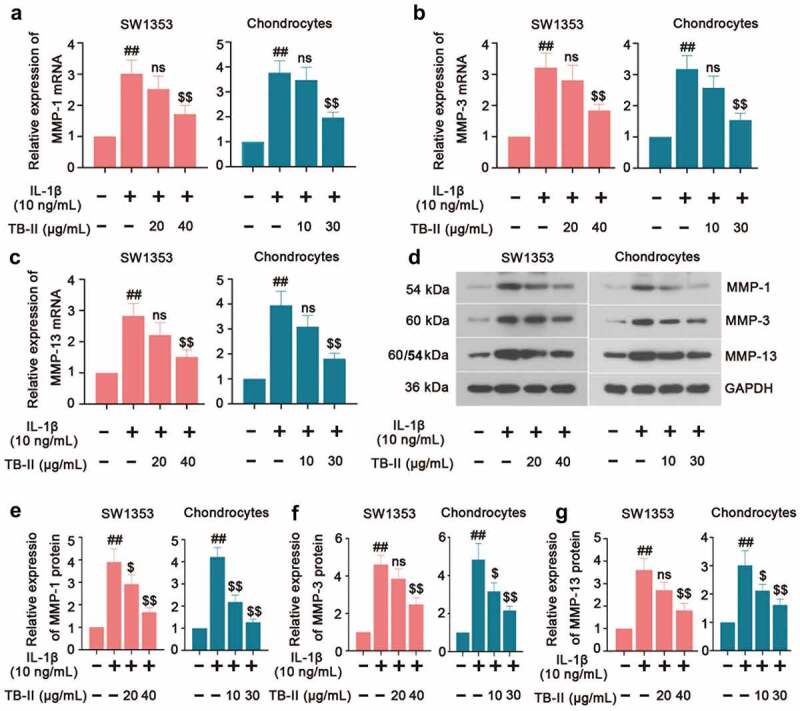
**TB-II inhibited the production of cartilage degrading enzymes in IL-1β-treated SW1353 cells and chondrocytes**. The mRNA expression of (a) MMP-1, (b) MMP-3 and (c) MMP-13 were detected by qRT-PCR. (d-g) The protein level of MMP-1, MMP-3, and MMP-13 were detected by Western blot. GAPDH was conducted as a loading control. One-way ANOVA, ^##^p < 0.01, compared with control cells; ^$^p < 0.05, ^$$^p < 0.01, ns: not-significant, compared with IL-1β-treated cells.

The imbalance between the anabolism and catabolism of the ECM is of great importance to OA development. Therefore, we further investigated the expression of the Matrix metalloproteinasesMMPs (MMP-1, MMP-3, and MMP-13) which play a crucial role in enzymatically digesting cartilage ECM components. In our study, qRT-PCR results showed an increased expression of MMP-1, MMP-3, and MMP-13 in IL-1β-induced SW1353 cells and chondrocytes, while it was reversed by TB-II treatment (Figure 4a-c). Meanwhile, Western blot assay exhibited the same trend of MMP-1, MMP-3, and MMP-13 expression (Figure 4d-g) as the qRT-PCR results. These data demonstrated that TB-II reduced the production of MMPs thereby preventing ECM degradation in IL-1β-stimulated SW1353 cells and chondrocytes.

### TB-II inhibited MAPKs and NF-κB signaling pathways in IL-1β-stimulated SW1353 cells

**Figure 5.**
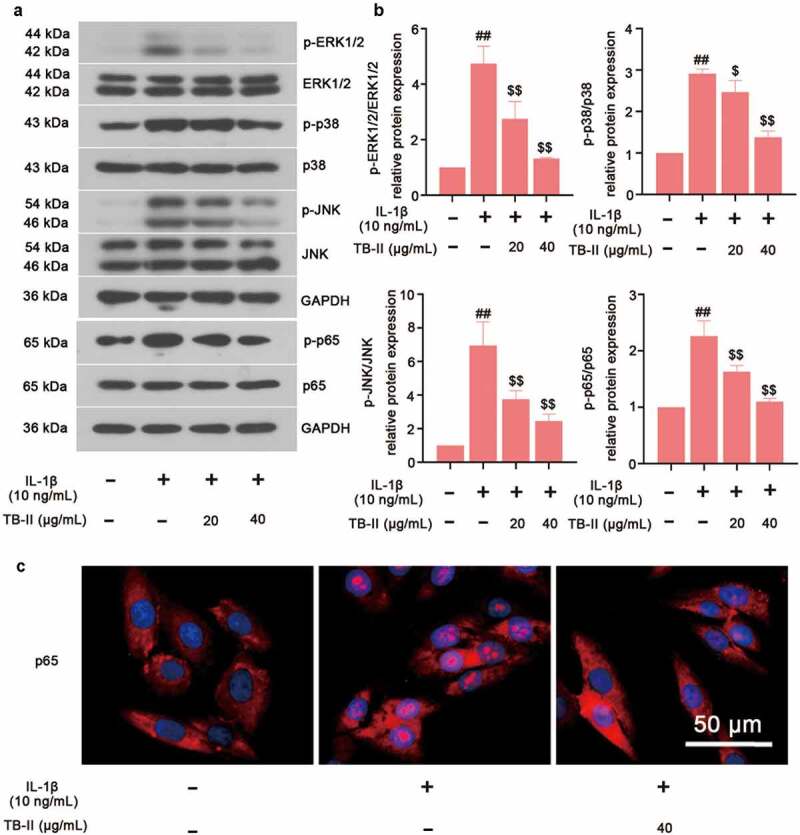
**TB-II inhibited MAPK and NF-κB signaling pathways in IL-1β-treated SW1353 cells**. (a, b) The protein expression of p-p65, p65, p-ERK1/2, ERK1/2, p-p38, p38, p-JNK, and JNK were determined by Western blot and quantified. GAPDH was conducted as a loading control. (c) The nuclei translocation of p65 (red) was detected by the immunofluorescence assay. Nuclei (blue) were stained with DAPI. One-way ANOVA, ^##^p < 0.01, compared with control cells; ^$^p < 0.05, ^$$^p < 0.01, compared with IL-1β-treated cells.

To explore the molecular mechanism of TB-II protecting against OA, the key proteins expression of MAPKs and NF-κB signaling pathways were examined. Western blot assay exhibited the up-regulated phosphorylation of ERK1/2, p38, JNK and p65 after IL-1β stimulation, indicating the activation of MAPK and NF-κB signaling pathways (Figure 5a and b). However, TB-II treatment significantly inhibited the phosphorylation of ERK1/2, p38, JNK and p65 (Figure 5a and b). Meanwhile, the translocation of p65 was shown in Figure 5c. Results showed that pretreatment with TB-II (40 μg/mL) repressed the translocation of p65 from the cytoplasm to the nucleus in IL-1β-stimulated SW1353 cells (Figure 5c). Taken together, these findings indicated that TB-II suppressed MAPKs and NF-κB signaling pathways.

## Discussion

**Figure 6.**
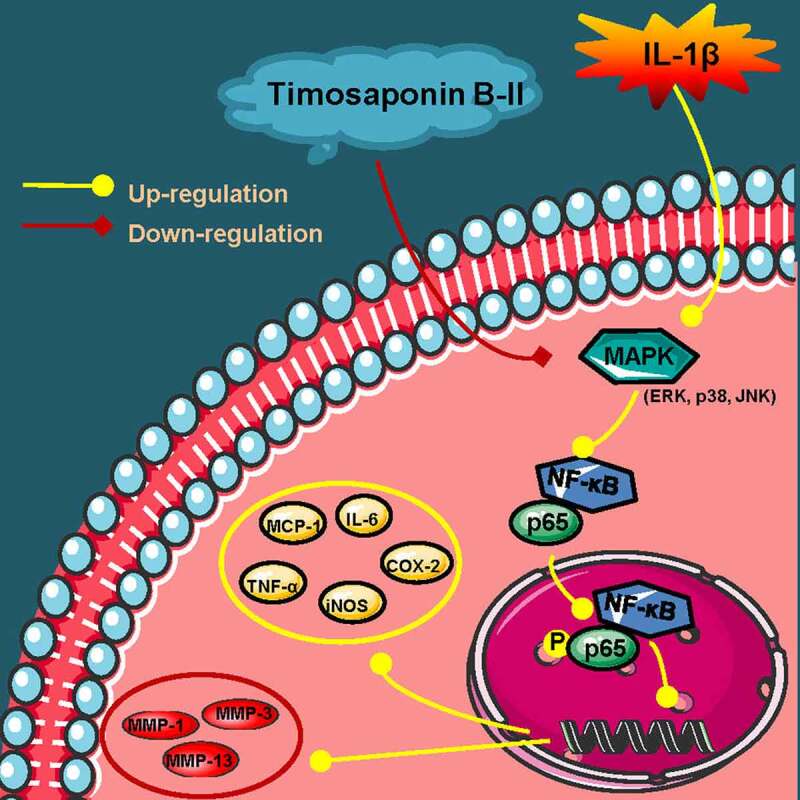
**A schematic diagram of in vitro anti-OA effects of TB-II**. Yellow arrows: A series of reactions of chondrosarcoma SW1353 cells stimulated by IL-1β. Red arrows: the mechanisms of TB-II in suppressing IL-1β-induced inflammation and ECM degradation in chondrosarcoma SW1353 cells. Yellow circle: inflammatory cytokines. Red circle: cartilage degrading enzymes.

OA is an age-related joint disease featured by the distraction of cartilage, which is common in the elderly. Since cartilage is non-regenerative, it will be worn out through the overloaded use of joints. Moreover, the inflammatory responses will be caused by friction between cartilage tissues, which can promote the degradation of ECM and ultimately lead to OA. However, most drugs implied to OA treatment include NSAIDs, acetaminophen, and duloxetine show serious side effects such as renal and gastrointestinal toxicity. Some studies highlight key genes involved in the progression of OA disease. Zhao et al. have found that enhancement of dual-specificity phosphatase 14 (DUSP14) limits OA progression by alleviating chondrocyte injury, inflammation and metabolic homeostasis [20]; Long et al. have found that MIR22HG inhibition ameliorates IL-1β-induced apoptosis and ECM degradation of human chondrocytes through miR-9-3p/ADAMTS5 pathway [21]. Besides, it is necessary to develop safe and effective drugs for specific OA treatment. Natural products have become the source of a large proportion of drugs. TB-II is a main bioactive compound in herb Rhizoma Anemarrhenae, exerting multiple pharmacological actions including anti-oxidative, anti-inflammatory and neuron – protective effects [22]. Herein, our study showed that TB-II exerted an anti-inflammatory effect and reduced the production of MMPs (MMP-1, MMP-3, and MMP-13) in IL-1β-stimulated SW1353 cells and chondrocytes (Figure 6).

Human chondrosarcoma cell-line SW1353 is widely used as a substitute for primary adult articular chondrocytes, however SW1353 cells cannot completely replace primary chondrocytes. However, we focus our attention on the inflammation and ECM degradation. We also should consider the difference in gene expression between SW1353 and primary OA chondrocytes after treatment with IL-1β [23,24]. Thus, we also used primary rat chondrocytes. Inflammation, a powerful catalyst for OA development, accelerates cartilage catabolism by secreting pro-inflammatory factors to induce ECM degradation, destroying the integrity and function of cartilage. Inhibiting inflammatory mediators induced by IL-1β can slow the progress of OA and alleviate the loss of proteoglycans. In addition, the pro-inflammatory factor IL-6 can inhibit the transcription and translation of COL2A1 by binding to sIL-6 R, thereby inhibiting the synthesis of COL-II [25]. Another study has indicated that IL-6 can also promote collagenase synthesis and collagen degradation through its combined action with IL-1α [26]. Our results showed that TB-II significantly inhibited the production of IL-1β-induced NO, iNOS, PGE_2_, COX-2, IL-6, and MCP-1, indicating that TB-II exerted an anti-inflammatory effect on IL-1β-stimulated SW1353 cells and chondrocytes. In addition, the pro-inflammatory factor IL-1β can promote the release of cartilage degrading enzymes, especially MMPs, and accelerate the ECM degradation to promote OA progress [27]. MMP is a family of proteolytic enzymes, which can degrade almost *all components of the* ECM and destroy the joint structure [28]. Meanwhile, the products of ECM decomposition can stimulate synovial cells and further induce inflammation, thus forming a vicious circle of inflammation and ECM degradation, providing a suitable microenvironment for the progress of OA [28]. Several studies have shown that reducing the production of MMPs (MMP-1, MMP-3, and MMP-13) can protect the cartilage matrix from being degraded, thereby alleviating OA progress [7,29]. Drug treatment before IL-1β stimulation in chondrocytes or SW1353 cells is a common method to study OA in vitro. Thus, cells were with IL-1β after TB-II pretreatment. Whether the concentration of the TB-II we selected could affect the cell activity was unknown, thus, the inhibition rate of TB-II on the cells was calculated. After all, chondrocyte apoptosis is also an important change in OA. Our results showed that TB-II inhibited the production of MMPs in IL-1β-stimulated SW1353 cells and chondrocytes, which revealed that TB-II might become a potential drug to alleviate the progression of OA. Other traditional Chinese medicine ingredients play a similar role, such as irisin which has been reported to alleviate the reduced COL-II expression and increased MMP-13 expression induced by IL-1β in SW1353 cells [30].

The MAPKs and NF-κB signaling pathways are reported to participate in OA development [31–33]. Inflammatory cytokines such as IL-1β can activate the MAPKs (ERK1/2, JNK, and p38) signaling pathway, leading to the expression of genes related to catabolic and inflammatory events. For example, studies have shown that the activation of MAPKs and the promotion of NF-κB p65 subunit translocating to the nucleus result in an increased level of NO, iNOS, COX-2, and PGE_2_ in IL-1β-induced rat chondrocytes [34,35]. These evidences explain that TB-II might promote OA-related inflammatory cytokine levels through MAPKs pathway in our study. Additionally, MAPKs are regarded as the main system of the cartilage matrix synthesis and MMPs production, which are involved in the degeneration of chondrocytes [36]. ERK1/2 inhibition can reduce the release of MMP-3 and MMP-13 in human chondrocytes treated with IL-1β [37]. Moreover, the MAPKs activation increase the expression of transcription factors such as RUNX-2, HIF-2α, and C/EBPβ, thereby further activating the NF-κB signaling pathway [31,38,39]. After activation, NF-κB p65 subunits are translocated to the nucleus and participate in regulating the expression of matrix-degrading enzymes and pro-inflammatory factors, affecting the quantity and remodeling of ECM. A study has confirmed that NF-κB activation can induce NO, iNOS, PGE_2_ and COX-2 production to promote the progression of OA [40]. Thus, it can be considered that inhibition of NF-KB signaling activation pathway is one of the important mechanisms of TB-II protecting against OA in this study. Previous studies have suggested that TB-II can exert an anti-inflammatory effect via suppressing the MAPKs and NF-κB pathways, supporting our results [11]. In addition, it is worth noting that we found TB-II reduced the IL-1β-induced ROS level. Recent research has shown that ROS and NF-κB signaling pathways interact to regulate cartilage metabolism and synovial inflammation, and play a unique role in the pathogenesis of OA [41]. Hence, our results further confirmed that TB-II might treat OA through antioxidant function.

Although our study provides some new clues to the function of TB-II in OA progression, there are some limitations exist. First, we only established a cellular OA model that could not completely mimic the characteristics of human OA. Second, the effects of TB-II on OA for in vivo studies have not been validated. In the future, we need to establish OA rat model for investigating the biological activities of TB-II in vivo.

## Conclusion

Taken together, TB-II was found to inhibit the IL-1β-induced inflammation, oxidative stress and ECM degradation via repressing the MAPKs and NF-κB signaling pathways, indicating that TB-II might become a new therapeutic drug for OA.

## Supplementary Material

Supplemental Material
