# Supplementary material for: Timosaponin B-II alleviates osteoarthritis-related inflammation and extracellular matrix degradation through inhibition of mitogen-activated protein kinases and nuclear factor-κB pathways in vitro
Source: Bioengineered. 2022 Jan 30;13(2):3450–61. doi: 10.1080/21655979.2021.2024685 (PMC8973927; doi:10.1080/21655979.2021.2024685)
Supplement: Supplemental Material [file KBIE_A_2024685_SM9288.docx]

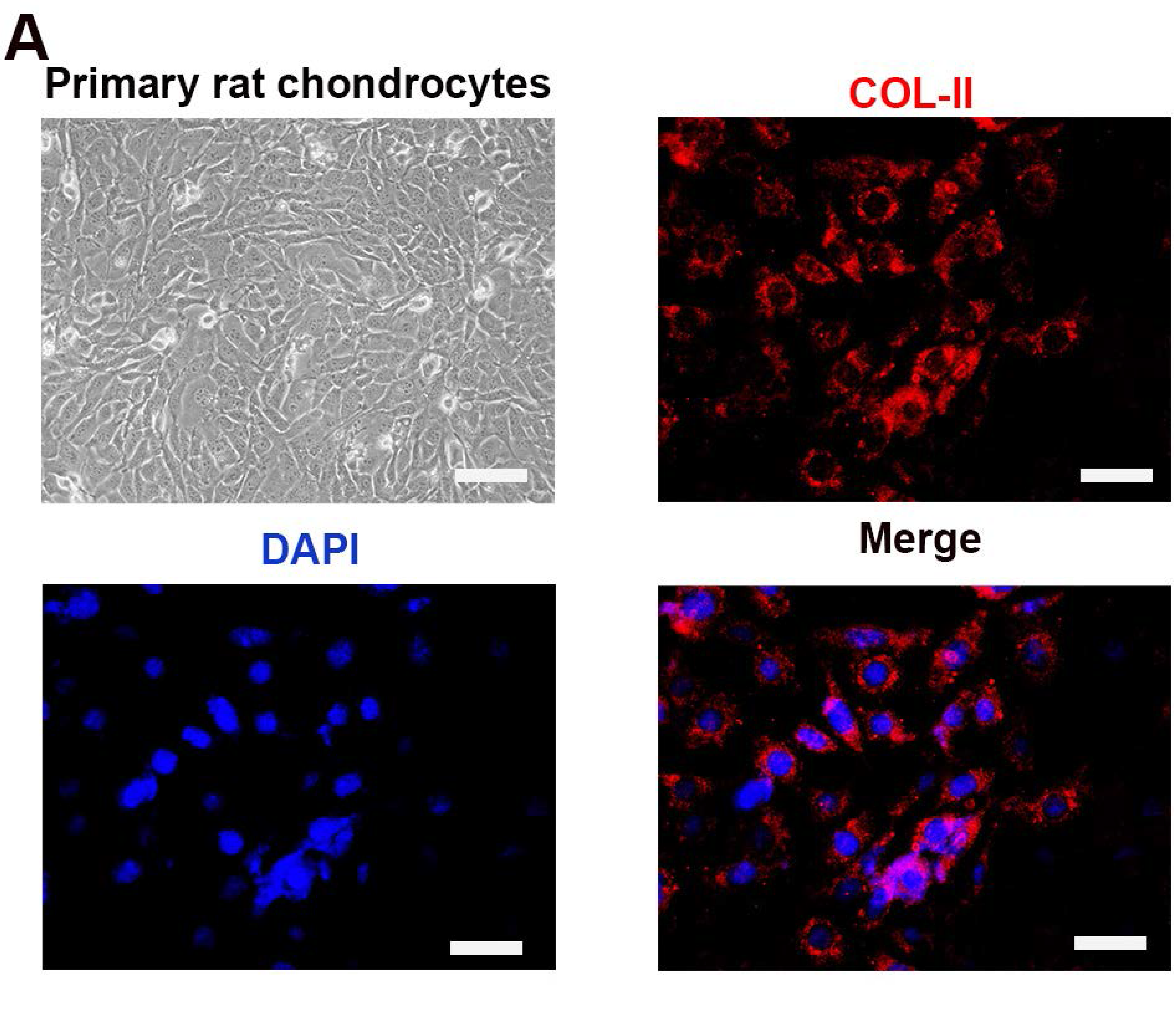


**Figure S1.** **The** **identification of chondrocytes.** (A) Morphology image and COL-II immunofluorescence staining of chondrocytes. Scar bar: 50 μm. COL-II (red) was detected by the immunofluorescence assay. Nuclei (blue) were stained with DAPI.
